# Whole Farm Net Greenhouse Gas Abatement from Establishing Kikuyu-Based Perennial Pastures in South-Western Australia

**DOI:** 10.3390/ani2030316

**Published:** 2012-08-03

**Authors:** Dean T. Thomas, Jonathan Sanderman, Sandra J. Eady, David G. Masters, Paul Sanford

**Affiliations:** 1 CSIRO Center for Environment and Life Sciences, Private Bag 5, Wembley, WA 6913, Australia; 2 CSIRO Sustainable Agriculture Flagship, St Lucia, QLD 4067, Australia; E-Mails: johnathan.sanderman@csiro.au (J.S.); sandra.eady@csiro.au (S.J.E.); david.masters@csiro.au (D.G.M.); 3 Department of Agriculture and Food Western Australia, 444 Albany Highway, Albany, WA 6330, Australia; E-Mail: paul.sanford@agric.wa.gov.au

**Keywords:** carbon sequestration, soil, sheep, grazing, methane

## Abstract

**Simple Summary:**

Greenhouse gas (GHG) emissions from ruminant livestock production (sheep, cattle and goats) have contributed to a common perception that a shift in the human diet from animal to plant-based products is environmentally responsible. In this study we found that the level of net emissions from livestock production systems is strongly influenced by the type of farming system that is used, and in fact GHG emission levels from some livestock production systems may be comparable with cropping systems. By introducing into farming systems ‘perennial’ pasture plants that are able to capture more atmospheric carbon, which is then stored in the soil, emission levels from livestock production can be substantially reduced.

**Abstract:**

On-farm activities that reduce GHG emissions or sequester carbon from the atmosphere to compensate for anthropogenic emissions are currently being evaluated by the Australian Government as carbon offset opportunities. The aim of this study was to examine the implications of establishing and grazing Kikuyu pastures, integrated as part of a mixed Merino sheep and cropping system, as a carbon offset mechanism. For the assessment of changes in net greenhouse gas emissions, results from a combination of whole farm economic and livestock models were used (MIDAS and GrassGro). Net GHG emissions were determined by deducting increased emissions from introducing this practice change (increased methane and nitrous oxide emissions due to higher stocking rates) from the soil carbon sequestered from growing the Kikuyu pasture. Our results indicate that livestock systems using perennial pastures may have substantially lower net GHG emissions, and reduced GHG intensity of production, compared with annual plant-based production systems. Soil carbon accumulation by converting 45% of arable land within a farm enterprise to Kikuyu-based pasture was determined to be 0.80 t CO_2_-e farm ha^−1^ yr^−1^ and increased GHG emissions (leakage) was 0.19 t CO_2_-e farm ha^−1^ yr^−1^. The net benefit of this practice change was 0.61 t CO_2_-e farm ha^−1^ yr^−1^ while the rate of soil carbon accumulation remains constant. The use of perennial pastures improved the efficiency of animal production almost eight fold when expressed as carbon dioxide equivalent emissions per unit of animal product. The strategy of using perennial pasture to improve production levels and store additional carbon in the soil demonstrates how livestock should be considered in farming systems as both sources and sinks for GHG abatement.

## 1. Introduction

The potential for Australia’s rural landscapes to store carbon is significant and could contribute in a substantial way to meeting national greenhouse gas reduction targets [1]. However, when considering the potential for sequestering carbon, flow-on effects that may negate the direct benefits of the new carbon store must be considered. In particular, there is a possibility of associated increases in emissions elsewhere which nullify or replace the abatement that would otherwise result from the activity [2]. Such a situation may arise if increased carbon sequestration is, for example, accompanied by increased numbers of grazing ruminants. Conversely, the increased emissions of such practice change, if considered in isolation, may also provide a misleading conclusion. For example, where ruminant livestock are considered primarily in terms of emission of greenhouse gases without appropriately accounting for the farming system in which they are produced [3]. A more relevant assessment should include net carbon storage within a farming system and/or greenhouse gas (GHG) emissions per unit of food energy or protein [4,5]. Therefore, it is essential to consider a whole farming system approach in assessing the net benefit of a particular carbon sequestration activity. 

Kikuyu-based pastures (*Pennisetum clandestinum* Hochst.) are being established in southern Australia to improve year-round feed supply in grazing systems and to increase water-use and ground cover to prevent soil degradation. Kikuyu is suited to the high rainfall zone (>600 mm annual rainfall) of south-western Australia, although growth may be limited in areas where frosts are frequent [6]. The high rainfall zone of south-western Australia comprises 3.7 million hectares of arable freehold land that is used for agriculture, of which about 20% is sown to crops each year [7]. Kikuyu pastures may also have a role in carbon sequestration because soil carbon has been found to increase in areas where perennial pasture species have been introduced into a predominantly annual species [8,9]. However, because feed supply is improved by establishing Kikuyu pastures it is likely that this change in farm practice may be associated with a concurrent increase in farm stocking rate and GHG (methane and nitrous oxide) emissions depending on how the additional feed supply is used in the grazing enterprise. Masters *et al*. [10] reported a 32% increase in optimal stocking rate when Kikuyu was established on 64% of the land allocated for winter grazing (pastures). Similarly, a case study by Omodei [11], found that stocking rate was 23% higher in paddocks with Kikuyu established. 

The aim of this study was to quantify changes in whole farm greenhouse gas emissions likely from the establishment of perennial grass-based pasture, so as to determine the net effects of the management change on the farm’s carbon balance.

## 2. Experimental Section

### 2.1. Farming System

A simulation experiment was conducted using the GrassGro biophysical model (version 3.2.2.) [12,13] to investigate the effect of establishing Kikuyu-based pastures on the farm biophysical environment, for a typical Merino sheep enterprise in south-western Australia. The GrassGro model is a grazing systems model, comprised of components that each describe a portion of the biophysical (climate, soils and land management units (paddocks), pastures, livestock), managerial (e.g., stocking rate, soil fertility, pasture grazing rotations and animal reproductive management) and financial subsystems, which form the ‘farm system’ under consideration [13]. These components combine to simulate biophysical and economic performance within the farm system at daily time steps for the chosen time interval (years), and from this data output summaries are generated using reporting templates, which can be customised. The simulation experiment was based on the Gnowellen location in Western Australia (34°24'S, 118°36'E), which is 94 km north-east of the city of Albany and corresponds with one of the experimental sites where soil carbon data was collected as part of the Australian National Soil Carbon Research Programme [14]. The farming enterprises in this region are characterised by a mixture of predominantly self-regenerating annual pastures and cropping.

Models were built for two farm systems:

1. Business-as-usual without the establishment of Kikuyu pasture, “Current”
2. Kikuyu pastures established on land that was allocated to pasture, “Improved”

These model scenarios were largely based on production and management parameters for a mixed cropping and self-replacing Merino sheep enterprise in the Albany Eastern Hinterland in Western Australia [10]. In the paper by Masters *et al*. [10], land allocations and livestock management parameters for farms with annual pastures only, and with a combination of annual pasture and Kikuyu are described, using the MIDAS bioeconomic optimisation model [15]. Based on Masters *et al.* [10], a land allocation of 70% annual pasture and 30% crop was selected for the Current farm and 45% Kikuyu pasture, 25% annual pasture and 30% crop for the Improved farm. Therefore, the Current and Improved model scenarios could be set up in GrassGro with either 2 or 3 paddocks available for grazing, respectively. Masters *et al*. [10] reported that the most profitable stocking rate for the two farm models was 8.1 and 10.7 dry sheep equivalents (DSE) per winter grazed pasture ha for Current and Improved farms, respectively, and these values were used in the simulation experiment. Winter grazed pasture area refers to the area of the farm that is retained for grazing (not cropped) during the winter/spring growing season. Common practice for the annual pasture areas in south-western Australia is to have rotations of crops and self-regenerating improved pastures on this land. Pasture swards are re-established infrequently. 

A validated plant model for Kikuyu was not available, so this was replaced with a different perennial grass (perennial ryegrass (*Lolium perenne* L.)) and combined with subterranean clover (*Trifolium subterraneum* L.) to create a perennial pasture in the GrassGro plant component. A combination of annual ryegrass (*Lolium rigidum *Gaud.) and subterranean clover was selected from the plant component for annual pastures. A sensitivity analysis was conducted to compare the effect on methane emissions of using different perennial grass plant models, including a developmental (beta) version of a Kikuyu plant model. The same yellow-grey duplex soil, reference Dy5.81 [16], was selected in the soil component for all paddocks in the model. 

In the GrassGro livestock component we selected the “Large Merino” breed. The weight of a mature ewe in average condition was set at 60 kg, with an annual greasy fleece production of 6.0 kg and average fibre diameter 21.0 µm. Ewe lambing occurred on 1 July each year and lambs were weaned on 1 September. Lambs were fed an 80:20 whole oats and lupin grain ration as required to reach a marketable weight of 45 kg by 31 December, at the latest, and were sold between 1 November and 31 December when they reached the required weight. The time of sale varied from year to year according to pasture conditions. A proportion of ewe lambs were retained each year to replace older ewes and stock mortalities. Flock ewes were supplemented with wheat grain to maintain body condition score greater than 2.5 and mature ewes were sold in the year that they reached 6 years of age. A shearing event was implemented on 30 October each year, to harvest wool from all mature sheep. 

All simulations were run over the years 1900–2010 using historical weather data for the experimental location obtained as Patched Point Datasets from the SILO database [17]. 

### 2.2. Soil Carbon

The annual rates of soil carbon change with adoption of Kikuyu-based pastures used in this study were derived from surveys conducted on five properties with established Kikuyu pastures in the south-west of Western Australia [18]. Kikuyu pastures ranged from 3 to 16 years in age. Soils at these sites were dominated by deep, well drained, moderately acidic, Yellow-Orthic Tenosols [19] typically with less than 7% clay throughout the upper 30 cm. In each of the Kikuyu pastures and an adjacent annual pasture (these were predominantly subterranean clover-based with volunteer annual forbs and grasses) eight randomly placed soil cores over an approximately 4 ha area were collected in three depth increments (0–10, 10–20 and 20–30 cm). Adjacent study sites were selected to be comparable to the Kikuyu pasture prior to the establishment of Kikuyu). A reconnaissance survey was conducted prior to sampling to ensure that adequate pairings were available for each Kikuyu-based pasture.

For carbon analysis, soil samples were first oven dried at 40 °C for 48 hours. Organic carbon concentration of the mineral soil (*c*, mg C g soil^−1^) was determined by dry combustion (LECO CNS2000, LECO Corporation, MI, USA) after sieving soils to <2 mm and then removing visible roots. Bulk density (ρ_s_, t m^−3^) was measured for each sample by use of volumetric rings to retrieve the samples. Soil organic carbon mass (*C_S_*, t ha^−1^) was then calculated as the product of *c* and ρ_s_ adjusted for the gravel content (*g*) and soil depth (*d*, m):

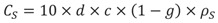
(1)

The annual rate of change in soil carbon stocks resulting from Kikuyu pasture establishment (*C_δS_*, t CO_2_-e ha^−1^ yr^−1^), was determined for each pasture:

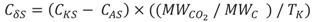
(2)
where

*C_KS_* is stored soil carbon in the Kikuyu pasture (t C ha^−1^)
*C_AS_* is stored soil carbon in the comparable annual pasture (t C ha^−1^)
*MW_CO2_* and *MW_C_* are the molecular weight of CO_2_ (44.0) and C (12.0), respectively
*T_K_* is the time since the Kikuyu pasture was established (years)

A one-sample *t*-test (one-tailed) was used to assess whether the rate of increase in soil carbon in Kikuyu pastures was greater than zero. 

### 2.3. Methane Emissions

Biophysical values from the GrassGro simulation model (Table 1) were used to calculate livestock methane during spring, summer, autumn and winter using the Sheep Greenhouse Accounting Framework V4 [20]. This accounting framework is based on methods described in the National Greenhouse Gas Inventory [21]. The Sheep Greenhouse Accounting Framework V4 is a spreadsheet model that uses livestock enterprise details (e.g., livestock class, stocking rate, feedbase quality) to produce a greenhouse gas emissions profile for a particular farm. The model breaks down greenhouse gas emissions into the various sources and where they are derived on the farm. The sheep liveweight values we applied to this model (Table 1) are estimates of total sheep body weight (including fleece, conceptus and seasonal structural changes such as stock reclassification), while the liveweight gain data are fleece and conceptus-free body gain estimates. 

Additional methane (*C_δM_* t CO_2_-e pasture ha^−1^ yr^−1^) produced in the Improved farm was calculated:

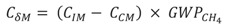
(3)
where

*C_CM_* is the methane produced in the Current farm (t CH_4_ pasture ha^−1^ yr^−1^)
*C_IM_* is the methane produced in the Improved farm (t CH_4_ pasture ha^−1^ yr^−1^)
*GWP_CH4_* is the global warming potential of methane (21 CO_2_-e, 100 year time horizon [22])

### 2.4. Nitrous Oxide Emissions

Biophysical values for both Current and Improved farms (Table 1) were used to calculate total daily nitrous oxide emissions attributable to livestock using the Sheep Greenhouse Accounting Framework.

**Table 1:** Biophysical values used as input values for the Sheep Greenhouse Accounting Framework for both Current and Improved farms taken from the GrassGro simulation model.

		Stocking rate	Liveweight	Liveweight gain^#^	Dry Matter Availability	Lambing Rates	Forage Protein	Forage Digestibility
		(DSE/pasture ha)	(kg/animal)	(kg/day)	(tonnes/hectare)	(% of ewes lambing)	(% CP*)	(% DMD**)
*Current farm — No Kikuyu established*								
Maiden Ewes	Spring	1.8	63	0.04	2.0	0.00	15	73
	Summer	1.8	58	−0.06	2.0	0.00	14	68
	Autumn	1.8	60	0.03	2.0	0.00	13	67
	Winter	1.8	59	0.11	2.0	0.00	15	75
Mature Ewes	Spring	6.3	69	0.03	2.0	0.75	15	73
	Summer	6.3	61	−0.11	2.0	0.00	14	68
	Autumn	6.3	60	0.00	2.0	0.00	13	67
	Winter	6.3	64	0.06	2.0	0.25	15	75
*Improved farm — 45% Kikuyu established*								
Maiden Ewes	Spring	2.4	62	0.03	2.0	0.00	14	71
	Summer	2.4	56	−0.06	2.0	0.00	13	67
	Autumn	2.4	58	0.04	2.0	0.00	14	68
	Winter	2.4	58	0.11	2.0	0.00	15	75
Mature Ewes	Spring	8.3	67	0	2.0	0.75	14	71
	Summer	8.3	58	−0.11	2.0	0.00	13	67
	Autumn	8.3	57	0.03	2.0	0.00	14	68
	Winter	8.3	62	0.07	2.0	0.25	15	75

^#^ Liveweight gain is fleece and conceptus free body weight gain * Crude Protein ** Dry Matter Digestibility

V4 as described previously for methane. Additional nitrous oxide (*C_δN_* t CO_2_-e pasture ha^−1^ yr^−1^) produced in the Improved farm was calculated:

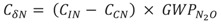
(4)
where

*C_CN_* is the nitrous oxide produced in the Current farm (t N_2_O pasture ha^−1^ yr^−1^)
*C_IN_* is the nitrous oxide produced in the Improved farm (t N_2_O pasture ha^−1^ yr^−1^)
*GWP_N2O_* is the global warming potential of nitrous oxide (310 CO_2_-e, 100 year time horizon [22])

### 2.5. Net Greenhouse Gas Emissions and Farm Production Efficiency

The value of establishing Kikuyu pasture as a carbon offset (GHG emissions abatement value) was calculated for winter grazed farm area (*OV_WG_* t CO_2_-e pasture ha^−1^ yr^−1^) or whole farm area (*OV_WF_* t CO_2_-e farm ha^−1^ yr^−1^):

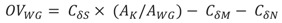
(5)

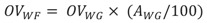
(6)
where

*A_K_* is the proportion of winter grazed (pasture) farm area that has Kikuyu established
*A_WG_* is the proportion of the farm that is allocated to winter grazed pasture

Meat and wool production efficiency both in terms of production per unit of land and production per unit of CO_2_-e were calculated using livestock and GHG production outputs from the GrassGro simulation model. Emissions were allocated either to meat or wool production based on the value of farm-gate sales revenue from each product class using 2004–2008 mean commodity prices reported by Australian Bureau of Agricultural Resource Economics [23]. 

## 3. Results

Soil carbon stocks provided by [18] indicated that there was significantly more SOC under Kikuyu pastures than in adjacent annual pastures (*P* = 0.016), with soil carbon accumulating at an estimated rate of 1.79 ± 0.55 t CO_2_-e ha^−1^ yr^−1^ in the Kikuyu pastures. Across the five paired sites, *Cs* (±1 s.e.) was 28.1 ± 5.1 t ha^−1^ in the Kikuyu pasture soils compared with 22.6 ± 4.2 t ha^−1^ in adjacent annual pasture soils (Table 2). There was a weak positive trend (*P* = 0.124) for a quadratic relationship between years since establishment and carbon accumulation rate. However, because this was not significant at *P* < 0.05 the mean carbon accumulation rate of all five sites (0.49 ± 0.15 t C ha^−1^ yr^−1^) was used in subsequent calculations and modelling. In the whole farm model this increase in soil carbon is equivalent to 0.80 t CO_2_-e farm ha^−1^ yr^−1^ sequestered, when Kikuyu pasture was established on 45% of the land.

**Table 2:** Soil organic carbon (SOC) in annual and perennial-based pasture paddocks at five survey sites in the south-west of Western Australia (taken from [18]).

		Annual Paddock		Perennial Paddock				
Survey site	Years since conversion*	Mean	95% CI**	Mean	95% CI	Total SOC increase	Annual SOC increase	
		t C ha^−1^		t C ha^−1^		t C ha^−1^	t C ha^−1^ yr^−1^	t CO_2_-e ha^−1^ yr^−1^
1	3	14.35	2.52	14.40	1.84	0.05	0.02	0.06
2	7	21.68	2.61	24.54	2.75	2.85	0.41	1.49
3	11	13.70	1.26	23.79	2.35	10.09	0.92	3.36
4	12	36.06	2.03	44.15	8.18	8.10	0.67	2.47
5	16	27.08	1.56	33.83	2.38	6.75	0.42	1.55
*Mean*	*9.8*	*22.57*		*28.14*		*5.57*	*0.49*	*1.79*

* The time since Kikuyu pasture was established in the paddock. ** Confidence Interval

Although there were some differences in pasture quality (metabolisable energy content) associated with differences in pasture species composition between the two farm models, greenhouse gas emissions produced per animal were comparable in both Current and Improved farm models. Due to a higher stocking rate in the Improved farm model, an additional 0.250 t CO_2_-e pasture ha^−1^ yr^−1^ of methane and 0.023 t CO_2_-e pasture ha^−1^ yr^−1^ nitrous oxide (a total increase of 0.273 t CO_2_-e pasture ha^−1^ yr^−1^ (equivalent to 0.19 t CO_2_-e farm ha^−1^ yr^−1^) as livestock GHG emissions) was produced. Values for soil carbon storage, and livestock emissions for Current and Improved farms are reported in Table 3. The net GHG emissions abatement value of establishing Kikuyu pasture in the Improved farm model was determined as 0.88 t CO_2_-e pasture ha^−1^ yr^−1^ and 0.61 t CO_2_-e farm ha^−1^ yr^−1^ (Table 3). Carrying higher stocking rates concurrently with a net reduction in farm greenhouse gas emissions resulted in a marked improvement in the efficiency of meat and wool production both in terms of production per unit of land and GHG emissions per unit of product (Table 3). 

**Table 3:** Carbon balance and livestock production efficiency of Current and Improved farm systems. Livestock production values for both Current and Improved farms were taken from the GrassGro simulation model.

Farm model	Current	Improved	Difference
Soil carbon storage (t CO_2_-e farm ha^−1^ yr^−1^)	0.00	0.80	0.80
Livestock methane emissions (t CO_2_-e farm ha^−1^ yr^−1^)	0.67	0.84	0.17
Livestock nitrous oxide emissions (t CO_2_-e farm ha^−1^ yr^−1^)	0.07	0.09	0.02
Net greenhouse gas emissions (t CO_2_-e farm ha^−1^ yr^−1^)	0.74	0.13	−0.61
			
Meat production (kg liveweight farm ha^−1^ yr^−1^)	92	118	26
Wool production (kg clean fleece farm ha^−1^ yr^−1^)	17.3	22.9	5.6
Meat GHG intensity (t farm CO_2_-e t liveweight^−1^)	4.2	0.6	−3.6
Wool GHG intensity (t farm CO_2_-e t clean fleece^−1^)	20.8	2.8	−18.0

**Figure 1:**
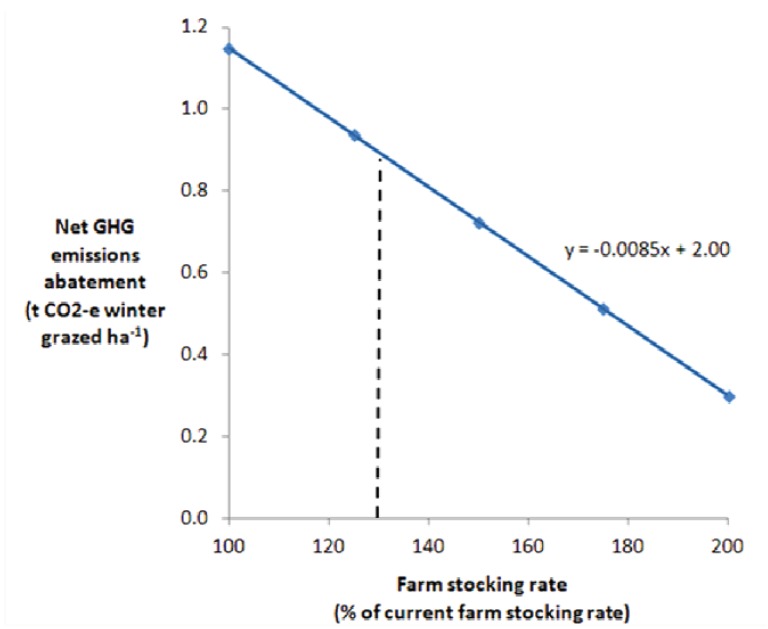
Relationship between farm stocking rate and the greenhouse gas (GHG) emissions abatement value of establishing Kikuyu pasture in place of annual pasture, based on a linear increase in farm GHG emissions with soil carbon storage kept constant. The vertical checked line indicates the likely change in farm stocking rate based on MIDAS bioeconomic modelling [10].

Effects of different changes to stocking rate coinciding with the establishment of Kikuyu on 45% of arable farm land are reported in Figure 1. This figure indicates that establishing Kikuyu pasture is likely to have a positive GHG emissions abatement value even if the farm stocking rate is doubled (at double normal stocking rate the net abatement value would be 0.30 t CO_2_-e pasture ha^−1^ yr^−1^), although this scenario is not expected because the business is likely to become less profitable. The model predicts that the value of establishing Kikuyu at the proposed level as a carbon offset would be reduced by 0.085 t CO_2_-e pasture ha^−1^ yr^−1^ for every 10% increase in farm stock numbers (Figure 1).

A sensitivity analysis using alternative perennial plant models within the GrassGro model showed that there was little effect on methane emissions and the subsequent net GHG emissions abatement values (Table 4). 

**Table 4:** Sensitivity analysis of methane emissions and net greenhouse gas (GHG) emissions abatement value with the inclusion of alternative perennial pasture species in the GrassGro simulation model

Perennial pasture species	Methane emissions	Net abatement value
	t CO_2_-e farm ha^−1^ yr^−2^	t CO_2_-e farm ha^−1^ yr^−1^
Perennial ryegrass	1.21	0.613
Kikuyu (beta version)	1.16	0.649
Lucerne	1.22	0.606
Phalaris	1.19	0.629

## 4. Discussion

### 4.1. Livestock Systems and Greenhouse Gas Emissions

The results of this study indicate that southern Australian agricultural systems that include livestock and use perennial plant-based permanent pastures may have substantially lower net GHG emissions, and reduced GHG intensity of production, compared with annual based production systems. Net farm carbon emissions were reduced by approximately 80% with the establishment of a perennial pasture plant (Kikuyu) compared with a livestock system based on annual pastures. Our finding is supported by a European grassland study where across 9 sites livestock-related emissions were equivalent to between 10 and 34% of the carbon sequestered in the soil [24]. Further, our results indicate that the net emissions from a perennial-based livestock system (where 86% of livestock GHG emissions were offset by sequestration of carbon in the soil) is likely to be less than, or at least comparable with, an annual cropping enterprise in terms of emissions per unit value of product [25]. Biswas *et al*. [25] report a carbon footprint value of around 0.3 kg CO_2_-e kg of grain^−1^, for crops produced in the central wheat belt of Western Australia. In the Improved livestock system in the current study the equivalent value for meat produced was 0.6 kg CO_2_-e kg liveweight^−1^. However, the farm-gate value of meat (per unit liveweight) is typically 6 or 7 times higher than grain. 

In view of this, the role of perennial plant based livestock enterprises for food and fibre production may be quite favourable in terms of its carbon footprint. This is additional to evidence that livestock production systems support higher soil carbon storage in their own right. A comprehensive review of data collected from 115 published studies by Conant *et al*. [26] concluded that the conversion of land from native land cover to well managed grazing (sheep and cattle) pastures generally increased soil carbon content. In contrast, land cleared for cereal cropping shows a decline in soil organic carbon initially before reaching a new equilibrium level [9]. The reduced net carbon emissions in the current study were accompanied by increases in stocking rate and production of wool and meat. As a consequence the GHG intensity of production of wool and meat when expressed as carbon dioxide equivalent emissions per unit of product decreased by more than 7 fold. This strategy of increased efficiency represents an effective approach to mitigation of methane emissions by livestock through a system of sustainable intensification [27,28], and would be effective until the rate of carbon accumulation in the soil plateaus. The GHG emissions abatement value reported in this study relies on the continued accumulation of carbon in the soil, so will likely start to decline at some point as the systems approaches a new steady state. The expected longevity of carbon capture by Kikuyu pastures has not been reported, however the IPCC suggest a default value of 20 years for such transitions following a change in management practice [22]. Other researchers consider this to be a conservative estimate and they reported net soil carbon storage in a number of permanent semi-natural grasslands [24]. 

### 4.2. Value of Kikuyu Pasture for GHG Emissions Abatement

The net GHG emissions abatement value when Kikuyu is established on 45% of farm land, taking into account leakage due to increased livestock numbers, was determined to be 0.61 tonnes CO_2_-e farm ha^−1^yr^−1^, for a mixed crop and livestock (30:70) property. Therefore, establishing Kikuyu pastures is likely to be a viable carbon offset practice, even when livestock numbers are increased concurrently (see Figure 1). However, changes in stocking rate with the establishment of Kikuyu pastures are likely to differ for different farm enterprises, due to individual farmer’s management choices and aversion to risk. Climatic differences between regions in the high rainfall zone of south-west Australia result in different duration and timing of feed deficits, so stocking rate responses vary accordingly. Furthermore, the 32% increase in optimal stocking rate determined using the MIDAS model [15] may be higher compared with realistic practices because farmers in this region tend to stock conservatively [29]. In farm systems where Kikuyu pastures are established primarily to provide out of season feed and reduce supplementary feeding, stocking rate (and therefore GHG emissions) may not increase as assumed in this model, and the GHG emissions abatement value would be higher than suggested (Figure 1). If stocking rates were increased above that included in this model, the abatement value would decrease at a rate of 0.085 t CO_2_-e pasture ha^−1^ yr^−1^ for every 10% increase in livestock numbers assuming that increased grazing intensity did not have an adverse effect on soil carbon accumulation rates. 

Clearly the net value of establishing perennial pastures as a carbon offset is closely linked to the rate at which additional soil carbon is stored, and this is influenced by a wide range of factors. Soil type, type and amount of fertiliser application, pasture species and rainfall are some of the factors affecting soil carbon accumulation [8]. However, other studies support the conclusion that the net carbon balance results reported for Kikuyu have a broader application outside of south-west Australia and probably apply to other perennial-based livestock systems. Chan and McCoy [30] found that soil under established Kikuyu pastures had a 30% higher soil organic carbon compared with setaria-based pastures. In another study, effects of pasture improvement (re-sowing and lime and P-fertiliser application) on soil carbon storage were examined [31]. In this study, a similar increase in soil carbon was reported for perennial and annual pastures. The lack of difference between perennial and annual pastures may have been due to the poor persistence of the perennial species, which was noted by the authors [31]. Based on a review of studies in a wide range of climatic zones and with different plant options, Post and Kwon [32] concluded that, on average, soil organic carbon accumulation in forest or pasture established after agricultural use was 33.8 g C m^−2^ y^−1^ and 33.2 g C m^−2^ y^−1^, respectively. The accumulation rates are similar to those derived from the current study (1.79 t CO_2_-e ha^−1^ yr^−1^ ~ 49 g C m^−2^ y^−1^, Table 2). In South America, deep-rooted grass pastures also increased soil carbon under grazing; much of this sequestered carbon was in the deeper part of the soil profile and would remain even through cropping rotations [33]. Many other studies now indicate that carbon sequestration in soils is much higher under permanent perennial-based pasture systems than annual cropping systems, e.g., [25,34]. While the benefits of perennial pastures look consistent, there is a strong case for more field studies that refine methods and measure the accumulation of soil carbon under a range of pasture scenarios in southern Australia. 

### 4.3. Estimating Farm Net GHG Emissions

Using GrassGro to adjust the NGGI default values for the sheep input parameters enabled the emission predictions to correspond with the region of interest for this study. The use of the dynamic biophysical model GrassGro has allowed an estimate to be made of the level of supplementary feeding required in each of the seasons, without which the appropriate feed quality parameters for estimating GHG emissions from livestock would not have been possible. Similarly additional outputs from the biophysical model allowed the calculation of carbon intensity of farm production, that is, units of production per unit of carbon emission. 

A Kikuyu model is currently being developed for GrassGro, but was not available for this simulation study. We used the perennial ryegrass plant model in place of Kikuyu in this study. It is likely that using a different plant model would have had some effects on the outputs of the simulation study. For example, Kikuyu tends to be more summer active compared with perennial ryegrass, so there may have been an increase in supplementary feeding in the Current model due to greater variability in seasonal feed supply. A sensitivity analysis showed that the overall effect of different plant models on methane emissions was relatively small. Animals were managed to maintain condition throughout the season through supplementary feeding, and therefore intake levels and bodyweight remained fairly consistent throughout the season (see Table 1). That is, systems where the sheep were eating a lower quality perennial grass and producing higher emissions per unit of energy intake, also required greater levels of supplementary feeding (which has lower emissions per unit of energy intake) to achieve production objectives. The use of a different perennial species in the GrassGro model had no bearing on the calculation of soil carbon accumulation, which was determined by an entirely independent field-based method. 

The role of establishing perennial pastures for the sustainability of agricultural systems is also influenced by a range of factors that were outside the scope of this study. In calculating the net GHG emissions abatement value for the modelled farm we have assumed that establishing Kikuyu based pastures requires no additional input of nitrogen fertiliser based on common farmer practice (anticipating that this can be provided by annual legumes that can persist in these pastures) [6]. However, if additional fertiliser is required this would need to be considered in the calculations of net GHG emissions, due to the release of nitrous oxide. In the farm system model a single enterprise structure was used, selected to represent our study area. However, differences in a range of sustainability indicators likely exist among different enterprise structures (e.g., [35]). Indeed, GHG emissions are only one of a diverse range of important sustainability indicators in agricultural systems. The impact of establishing perennial pastures on agricultural sustainability more broadly and for other enterprise structures still needs to be investigated. 

## 5. Conclusions

In this study we report the net GHG emissions for a livestock farming system where a perennial grass (Kikuyu) has been established. Net GHG emissions were determined by deducting leakages from introducing this practice change (increased methane and nitrous oxide emissions due to higher stocking rates) from the soil carbon sequestered from growing the Kikuyu pasture. Carbon sequestered in the soil outweighed the increase in other GHG emissions from increased stocking rates, and we determined that net carbon sequestration was 0.61 t CO_2_-e farm ha^−1^ yr^−1^ more than that calculated from an annual pasture based livestock system, while the rate of soil carbon sequestration in the perennial pasture is maintained. This resulted in a decrease in net greenhouse gas emissions from 0.74 to 0.13 t CO_2_-e farm ha^−1^yr^−1^. Based on likely changes in stocking rate associated with establishing Kikuyu pasture, we believe this is a conservative estimate. This study highlights the importance of considering the full GHG emissions implications of a change in management practices and may have wider implications for establishing perennial forage species in livestock systems. Provided other perennial species are able to accumulate soil carbon at a rate similar to Kikuyu, sowing perennial forage plants in areas where annual pastures or crops exist may be an important part of a greenhouse gas abatement strategy. 
